# Assembly and Channel Opening in a Bacterial Drug Efflux Machine

**DOI:** 10.1016/j.molcel.2008.02.015

**Published:** 2008-04-11

**Authors:** Vassiliy N. Bavro, Zbigniew Pietras, Nicholas Furnham, Laura Pérez-Cano, Juan Fernández-Recio, Xue Yuan Pei, Rajeev Misra, Ben Luisi

**Affiliations:** 1 Department of Biochemistry, University of Cambridge, 80 Tennis Court Road, Cambridge CB2 1GA, UK; 2 Life Sciences Department, Barcelona Supercomputing Center, Jordi Girona 29, Barcelona 08034, Spain; 3 School of Life Sciences, Arizona State University, Tempe, AZ 85287-4501, USA

**Keywords:** MICROBIO, PROTEINS

## Abstract

Drugs and certain proteins are transported across the membranes of Gram-negative bacteria by energy-activated pumps. The outer membrane component of these pumps is a channel that opens from a sealed resting state during the transport process. We describe two crystal structures of the *Escherichia coli* outer membrane protein TolC in its partially open state. Opening is accompanied by the exposure of three shallow intraprotomer grooves in the TolC trimer, where our mutagenesis data identify a contact point with the periplasmic component of a drug efflux pump, AcrA. We suggest that the assembly of multidrug efflux pumps is accompanied by induced fit of TolC driven mainly by accommodation of the periplasmic component.

## Introduction

Resistance to drugs is an obstacle in the treatment of infections by pathogenic bacteria. One mechanism of resistance stems from the activity of efflux transporters that displace a broad range of antibiotics and other toxic compounds from bacterial cells (Poole, 2001; Sulavik et al., 2001). In Gram-negative bacteria, these transporters take the form of three-component efflux pumps that span the inner and outer membranes and the interstitial periplasm. The pump comprises an outer membrane channel, an inner membrane protein that transduces electrochemical energy, and a bridging protein that links the transmembrane components through protein-protein interactions in the periplasm (Thanassi and Hultgren, 2000; Delepelaire, 2004). Analogous multicomponent pumps have been identified that export proteins such as toxins and adhesion factors, and for such pumps the inner membrane component is typically a member of the ATP-binding cassette transport family (Nikaido and Zgurskaya, 2001; Dawson and Locher, 2006). Crystal structures are available for representatives of all three components of tripartite efflux pumps (Koronakis et al., 2000; Murakami et al., 2002; Akama et al., 2004), but the structure of the ternary assembly is presently unknown.

**Figure 1:**
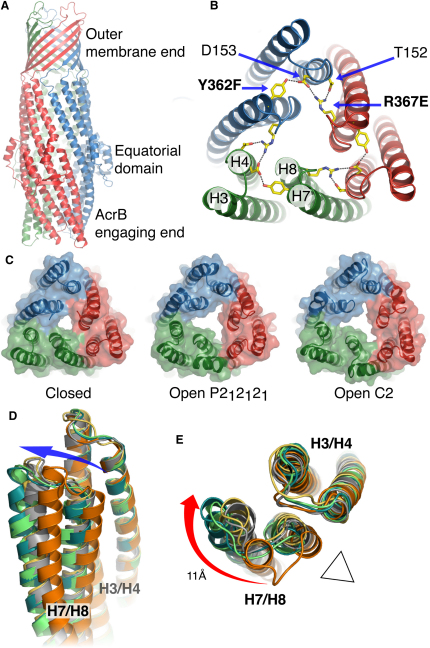
The TolC Outer Membrane Protein and Its Partial Opening (A) A side view of the TolC homotrimer (1EK9). (B) A schematic of the mutations that were studied here to stabilize channel opening. A network of charged interactions maintaining the resting closed state of TolC (1EK9). These include Y362, R367 from H7/H8, which are coordinated by T152, and D153 from H3/H4. Y362 has been mutated to F and R367 to E to disrupt the network of salt bridges (shown in bold). The protomers are colored red, blue, and green. (C) Crystal structures of the open and closed states. The view is along the trifold axis at the periplasmic, AcrB-engaging end of the TolC trimer. (D) Helical movements from the open to the closed state for the two crystal forms. Transition of the mobile helices H7/H8 from closed state (orange) to open as exemplified by the different subunits of C2 (cyan and green) and P2_1_2_1_2_1_ (gray and yellow). The view is of overlays of helical fragments H7/H8 (foreground) and H3/H4 (background), revealing the minimal relative movement of H3/H4 static helices as compared with H7/H8. (E) Top view of the same overlays shown in (D). The displacement of the H7 helix is up to 11 Å in the C2 structure. Note also the lagging of the H8 helices and the relative swing of the H7 in respect to H8. The triangle indicates the molecular trifold axis.

One well-studied efflux pump is the tripartite assembly formed in *E. coli* by the outer membrane protein TolC (Wandersman and Delepelaire, 1990; Fralick, 1996), the periplasmic protein AcrA, and the inner membrane protein AcrB, which uses proton motive force to translocate acridine and other drug-like compounds. TolC is a homotrimeric channel assembly with a transmembrane spanning portion and an extensive periplasmic domain that has two distinct sections: an outer membrane proximal subdomain in which 12 helices pack to form a hollow cylinder, and a distal subdomain where 12 helices form conventional coiled coils that curve to meet at a juncture that occludes the channel for all but the smallest ions (Figures 1A and 1C). The interaction of adjacent coiled-coil pairs is stabilized in part through hydrogen bonds and salt bridges, and mutation of the residues participating in these interactions causes the channel to become permissive to the diffusion of ions across the membrane in vitro (Andersen et al., 2002) and to the entry of antibiotic in vivo (Augustus et al., 2004).

In this study, we have examined a network of hydrogen-bonding interactions at the aperture end of the TolC which, when mutated, partially opens the channel so that it becomes leaky to bulky molecules in vivo. The crystal structures of the mutant reveal partially opened states and suggest how exposed grooves might engage the periplasmic component of the acridine efflux pump, AcrA. A model of the AcrA/TolC interaction successfully predicts a mutation pair in the proteins that compensate each other's disruptive effects, suggesting that K383 from TolC forms a functional salt-bridge interaction with D149 from AcrA, which is crucial for the assembly of a functional pump (for reasons of compatibility with previous publications, the residue numbering for TolC corresponds to the mature protein sequence lacking the 22 amino acid signal peptide, but for AcrA it corresponds to the precursor protein sequence). Using docking calculations, we propose how the open form of TolC intermeshes with the inner membrane component of the efflux pump, AcrB. The functional and structural data and computational models suggest how the multidrug efflux pump is assembled and how AcrA is accommodated through induced fit of intraprotomer grooves in TolC.

## Results

### Generation of a Leaky TolC by Mutagenesis

Inspection of the intersubunit interactions that maintain the closed state suggests the importance of a network of hydrogen bonds and salt bridges mediated by Y362 and R367 (Figure 1B) (Andersen et al., 2002). We prepared a double mutant to disrupt this network (Y362F and R367E) and found that expression of the protein in a *tolC* null strain of *E. coli* increased sensitivity to the antibiotic vancomycin, independent of AcrA or AcrB, suggesting that the mutation permits the passive diffusion of this large (1450 Da) antibiotic across the outer membrane (Table 1). Furthermore, the TolC double mutant conferred survival to a null strain lacking the LamB maltoporin when grown on maltodextrin as a sole carbon source, whereas bacteria expressing wild-type TolC did not grow under these conditions (data not shown). Nonetheless, the mutant protein retains the capacity of forming a functional efflux assembly, because it operates equally well as the wild-type TolC in conferring partial resistance to the antibiotic novobiocin that is extruded by the efflux pump composed of TolC, AcrB, and AcrA (Table 1). In control experiments, neither the mutant nor the wild-type TolC restores resistance to novobiocin in strains lacking either AcrA or AcrB.

### Crystallographic Analysis of the Leaky TolC Mutant Reveals Partial Channel Opening

We have solved the crystal structure of the Y362F, R367E mutant in two crystalline forms at 3.2 and 3.3 Å resolution (Figure 1C). The crystallographic data are summarized in Table 2. Corroborating the in vivo effects, the crystal structures of the double mutant reveal significant repacking of the helices in the distal helical domain that partially opens the channel, relative to the closed structure (Figures 1C–1E). Both structures also identify a putative chloride ion binding site formed by the peptide backbone at the junction of the transmembrane and periplasmic domains that is likely to play a role is stabilizing the conformation (see Figure S1 and Supplemental Data available online).

**Figure 2:**
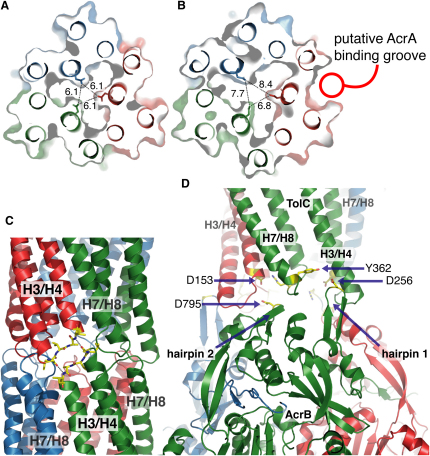
Potential Docking Faces of the Partially Opened State of TolC (A and B) The second aperture of the channel is less perturbed in the crystal structures of the partially opened state. A cross-section through the TolC channel in the closed-state (A) and open-state C2 crystal form (B) at the level of the second selectivity filter formed by the ring of D374 residues. While the outer opening of the channel (as measured by the position of G365) from the 8.5 Å in closed state to about 20 Å in the C2 form (Figure 1C, Figure S2), the interior second selectivity filter composed of a ring of D374 deeper in the channel is much less perturbed. Although the distance between the D374 is extended from about 6.1 Å in 1EK9 to up to 8.4 Å in the C2 form, it is unlikely to be sufficient for even small molecules to pass unimpeded, thus suggesting that a further opening of the channel is required for transport. This is likely to be activated by the engagement of the periplasmic partner protein, AcrA. Note the deepening of the predicted AcrA binding groove in the partially open C2 structure. (C) The intermesh of the loops in the packing of the TolC open state, showing details of the trimer-trimer contact interface across a crystallographic symmetry operation in the C2 crystal form. This interaction may mimic the docking of the TolC into the matching surface of the AcrB (shown in [D]). (D) Docking model of AcrB and a model of open-state TolC based on the C2 crystal structure. Colored by chain. The model of the AcrB-TolC complex was prepared using the asymmetric structures (2GIF and C2 crystal form of TolC). Although β2 hairpin is in proximity of H7/H8, it is still capable of interacting with the H3/H4 residues, in agreement with crosslinking data (Tamura et al., 2005). Residues indicated by arrows are D153, one of the residues included in TolC wild-type that maintains the closed gate; D795 from AcrB, a residue from AcrB β2 hairpin, which could potentially disrupt D153 interactions; and Y362 (another gating residue from TolC) and D256 (from β1 hairpin of AcrB), which in our refined docking model are close to the interface.

The TolC protomer comprises an imperfect structural repeat, and there are two sets of coiled coils at the distal end of the TolC that differ significantly in their superhelical trajectories in the closed resting state. The crystal structures of the double mutant show that three of the six pairs of coiled coils move radially outward from the central molecular axis of the trimer in comparison with the closed-state form, widening up the periplasmic lumen of the channel like the iris mechanism of a camera (Figures 1D and 1E). This movement occurs in the C-terminal pair of coiled coils for each of the protomers, corresponding to helices H7/H8 and associated loop 363–369. There is much less structural change in the N-terminal helical pair H3/H4 and associated loop 145–151. The channel remains partially occluded by a second aperture located further inside (Figures 2A and 2B). This second aperture is composed of a ring of D374, contributed from each protomer.

Superposition of the mutant structures with the closed-state crystal form reveals that the greatest conformational changes occur in the H7/H8 helices. The H3/H4 helices are comparatively static and are like a stator around which the H7/H8 pair swings, as illustrated in Figures 1D and 1E. The movement is associated with partial sheering of the H7/H8 coiled-coil interface.

In one of the crystal forms (spacegroup C2), neighboring TolC trimers pack tightly so that the exposed loop regions of the coiled coils H3/H4 and H7/H8 pair in a self-complementary fashion, and the H3/H4 loops form a hydrogen-bonding network (Figure 2C). This crystal form has a greater displacement of the helical pairs H7/H8 compared to the P2_1_2_1_2_1_ form. In the C2 form, the maximal displacement of the H7 helix on the leading edge is over 11 Å in the case of the chain B, and the corresponding maximal displacement of the lagging H8 helix from its original position in the resting state is roughly 4.5 Å (Figures 1D and 1E). In both crystal forms, the displacement of helices differs for each of the three subunits, resulting in an asymmetry (shown in Figure S2).

Although the movement of the helices in the TolC mutant structures provides sufficient opening for passage of molecules past the distal tip of TolC, the second aperature is still occluding. While it is being distorted in the observed structures and is likely to fluctuate into an open state, the second aperature is not fully disrupted, suggesting that further conformational adjustments are required for greater channel opening. This is likely to be driven through protein-protein interactions in the efflux pump assembly, to which we will now turn.

### Interactions of Open-State TolC with AcrB

The location of protein-protein interfaces in efflux pumps is becoming better defined from the accumulation of biochemical and mutagenesis data. Crosslinking experiments have identified residues in TolC and AcrB that are likely to be in proximity within the acridine efflux pump (Tamura et al., 2005), and some of these map onto the open state of TolC at the static loop (helices H3/H4). The pattern of crosslinks is consistent with an intermeshing of AcrB and TolC, but one in which the recognition elements, namely protruding loops and small canyons, permit only limited depth to the surface interpenetration. We note that a similar interpenetration takes place in the C2 crystal form of the TolC mutant, where H3/H4 helices pack against each other in symmetry-related molecules (Figure 2C). It is thus plausible that the shallow intermeshing of TolC trimers may mimic its interactions with AcrB in the acridine efflux drug assembly, where backbone-mediated interactions play a role in the engagement of AcrB and TolC static helices H3/H4 (Figure 2D).

Analysis of charge distributions on the periplasmic surfaces of TolC and AcrB reveals complementary electrostatic patterns. Docking of the asymmetric AcrB and the partially opened TolC structure shows that only two orientations display satisfactory surface complementarity, and energy calculations predict significantly better interaction for the model in which AcrB β hairpin 2 intermeshes in the interprotomer space formed by loops belonging to TolC helical pairs H3/H4 and H7/H8 (Figure 2D; Table S1). This is consistent with crosslinking data (Tamura et al., 2005), which indicate that residues from the β2 hairpin of AcrB lie in the vicinity of the H7/H8 region of TolC. Hairpin β1 engages the static helices H3/H4 in a side-chain-independent fashion, with a focal point at G147, a TolC residue that is prominently crosslinkable to the β1 hairpin of AcrB in studies by Tamura et al. (2005) and is also a contact point between the symmetry-related molecules in the C2 crystal form of the TolC mutant (Figure 2C). The proposed interaction does not require defined side-chain interactions, and, consistent with this model, efflux pump activity is comparatively insensitive to amino acid substitutions in this β1 hairpin region (F. Husain and R.M., unpublished data). Residues that are in proximity at the proposed AcrB/TolC interface are indicated in Figure 2D, and these include D153 and Y362 of TolC, which maintain the closed state of the channel.

It would appear that AcrB-TolC interaction should be sufficient for the extent of opening observed in the reported structures. This is further supported by the recent description of the crystal structure of the AcrB in complex with the transmembrane protein YajC, resulting in the introduction of a twist in the AcrB, which is compatible in handedness and rotation with our observed aperture-like opening of TolC (Tornroth-Horsefield et al., 2007). Further dilation of the channel, however, may require destabilization of the second selectivity filter, i.e., the inner ring formed by D374, and most likely this requires additional energy. This could be provided from a conformational change in AcrB itself; however, in all available structures of AcrB, the exposed hairpins that seem to be responsible for engaging TolC undergo the least conformational change, even in drug-bound states, and consistently have low overall crystallographic thermal disorder factors. The shallow interpenetration of the AcrB/TolC interface, and its anticipated conformational stasis with liganded-state AcrB, suggests that the interaction of inner and outer membrane proteins will only partially open the channel. Thus, the AcrA interactions may be key to the opening process.

### Interactions of the Open-State TolC and AcrA

In comparison with the previously reported closed structures of TolC, the mutant structures of TolC represent a partially opened state, which develops a shallow groove within the protomer that may accommodate AcrA (Figure 2B). This groove appears in all three protomers and extends from the periplasmic lumen of the TolC channel all the way up to the equatorial domain. The same region has been mapped by chemical crosslinking as the site of AcrA/TolC interaction (Stegmeier et al., 2006; Lobedanz et al., 2007) and the location of compensating mutations that confer AcrA/AcrB compatibility to the *Vibrio cholerae* TolC homolog (Vediyappan et al., 2006) and MexA/MexB compatibility to the *E. coli* TolC (Bokma et al., 2006). It is possible to dock the coiled-coil region of AcrA into the intraprotomer groove formed between the H3/H4 and H7/H8 helices. The structural rearrangement of the H7/H8 helices increases their solvent accessible surface from about 1000 Å^2^, as seen in the closed state (1EK9), to up to 1500 Å^2^ in chain B of the partially open-state C2 crystal form and also deepens the proposed AcrA binding groove (Figure 2B). Although AcrA is capable of accessing the closed state of TolC, the deepening of the groove is likely to favor the complex. Docking simulations reveal that the binding energy of the closed state is about half that of the most divergent subunit of the C2 form (−24.9 kcal/mol versus −51.4 kcal/mol). The hairpin in this orientation can form a salt bridge interaction with residues in the groove and the equatorial domain, accounting for effects of mutations in this region on activity (Augustus et al., 2004). Residues in these regions covary in evolution, which indicates that they may interact (data not shown). Previous work using chemical crosslinking has identified potential interacting residues on the surface of AcrA and TolC (Stegmeier et al., 2006; Lobedanz et al., 2007). Taking these data into account results in a single preferred orientation of the AcrA hairpin, in which several charged residues appear to be in proximity, most notably K383 from TolC and D149 from AcrA respectively (Figure S3).

To test our AcrA-TolC docking model, we examined the phenotypes of the mutants K383D (TolC) and D149K (AcrA). When compared with the wild-type proteins, the single mutants are hypersensitive to novobiocin, indicating functional inactivity of the efflux pump (Table 1). Thus, each of the mutations K383D (TolC) and D149K (AcrA) has a deleterious effect on the pump's activity, most likely by affecting its assembly. When both mutations have been introduced simultaneously, the pump's activity was reconstituted and may exceed that of the wild-type proteins (Table 1). Notably, the K383D mutation has no effect on the vancomycin sensitivity (Table 1), suggesting that it does not affect the leakiness of the channel.

The structural changes associated with opening of the TolC channel include modest conformational changes in the equatorial domain that lie at the junction of the two helical subdomains. Deletion of part of the equatorial domain impairs transport (Yamanaka et al., 2007), suggesting that it plays a role in the allosteric transition to the open state. One key residue in the equatorial domain is R390, and previous studies have shown that the mutation R390C gives a drug-sensitive phenotype (Augustus et al., 2004; Werner et al., 2003). We observe a similar phenotype for the R390E mutation (Table 1), which may make the channel leaky. It is thus likely that R390 is linked to aperture opening of the TolC channel. In the closed state, the aliphatic portion of the R390 side chain makes extensive hydrophobic interactions with residues F201 and V198 in the equatorial domain and L386 and I341 in the neighboring coiled-coil partner. The substitutions at R390 are likely to bias the structure of the open state by affecting the supercoiling of the coiled coils.

## Discussion

The crystal structures of a mutant TolC have allowed us to visualize the partially open state, and a crystalline lattice contact provides a model for how it intermeshes with AcrB, which is supported by docking calculations. The open-state structures reveal three intraprotomer grooves, and these are likely to engage with three copies of AcrA per TolC trimer in the periplasm (Fernandez-Recio et al., 2004; Lobedanz et al., 2007). Our model of AcrA/TolC docking successfully predicts mutations that compensate disruptive substitutions.

Recent crystallographic data show that AcrB is conformationally asymmetric, with three nonequivalent liganded states observed (Murakami et al., 2006; Seeger et al., 2006; Sennhauser et al., 2006). There appears to be corresponding asymmetry of TolC open structures, which is shown by crossprotomer distances (Figure S2). The asymmetry of the open-state structures is also reflected in the exposed surface of the three grooves that putatively engage AcrA. These differ by roughly 15% within the trimer, affecting also the predicted binding energies of AcrA-TolC interaction. They are particularly pronounced in the C2 crystal form, which also displays the largest opening of the channel. However, the structures of AcrB in the asymmetric state do not show a significant displacement of the protruding TolC-binding surface, which suggests that the pairwise interaction of TolC with AcrB may not be directly affected by the asymmetry during the transport process. The asymmetry of AcrB may result in nonequivalent interactions of AcrA with AcrB and TolC during the transport process.

Accumulating structural and functional data are helping to evolve ideas of how the components of tripartite efflux pumps associate and how their interactions drive TolC channel opening. Earlier models ascribed a key role to the inner membrane component in opening the TolC channel (Murakami et al., 2002). However, the possibility of a more active role for the periplasmic component in channel opening has been suggested (Mikolosko et al., 2006), although at the time no experimental evidence was available to test the hypothesis. Mutagenesis (Stegmeier et al., 2006; Vediyappan et al., 2006; Bokma et al., 2006; Nehme and Poole, 2007) and crosslinking studies (Lobedanz et al., 2007) indicate that there are extensive, well-defined interaction interfaces between the periplasmic and outer membrane proteins, and models for the organization of the periplasmic component in the efflux machinery have been suggested from several structure-based theoretical models (Higgins et al., 2004; Akama et al., 2004; Fernandez-Recio et al., 2004). We speculate that AcrA is more than just a passive bridge between the energized AcrB and TolC but also an active transducer of energy from one to the other. Despite the dramatic changes in the AcrB during its work cycle (Murakami et al., 2006; Seeger et al., 2006), the TolC-interacting interface changes little, suggesting that this interface might only partially transmit the conformational changes to TolC and permit incomplete opening of the channel (Murakami et al., 2006). The induced fit of the periplasmic partner into the partially opened grooves of TolC may complete the process of channel opening.

**Figure 3:**
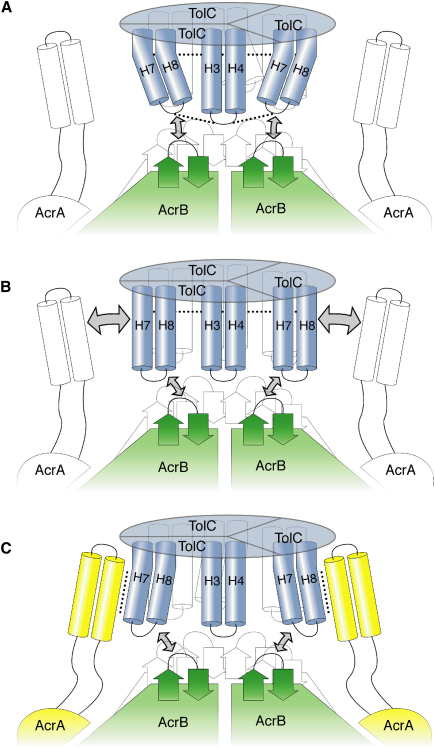
A Cartoon Schematic of the Assembly of the Efflux Pump and Accommodation of AcrA by TolC (A) AcrB docking of TolC results in intermeshing of the exposed crown and helical protrusions. “Mobile” H7/H8 helices surround one “static” helix pair H3/H4 in the middle. Two salt bridges (small dotted lines) support the closed state (as represented by the bent helices). Horizontal dotted line higher up in the TolC channel represents second selectivity filter. Interaction of the TolC H7/H8 helices with the β hairpins of the AcrB crown is represented with two-sided arrows. The AcrA (outlined) may be predocked on the AcrB. (B) Unlocking of bridges. Interaction of β hairpins 1 and 2 in the crown of AcrB with the helical turns of H7/H8 helices from TolC breaks the intramolecular salt bridges, releasing the H7/H8 helices and partially opening the iris of the outer periplasmic entrance of the TolC channel and deepening of the surface grooves allowing the engagement of the AcrA (indicated by two-sided arrows). This AcrB interaction, however, does not affect the second selectivity filter. (C) AcrA binds into the surface grooves of TolC, disrupting the second selectivity filter and opening the channel to its full extent. The machinery is now fully assembled and active for transport.

Combining our findings and other observations (Murakami et al., 2006; Stegmeier et al., 2006; Lobedanz et al., 2007; Nehme and Poole, 2007), we envisage two energetically distinct steps in the transport mechanism of the acridine efflux pump and related assemblies (Figure 3). In one step, the helical protrusions of TolC engage in the exposed periplasmic crown of AcrB, regardless of drug-bound state or energy state, resulting in partial opening of the TolC channel (Figures 3A and 3B). The preferred order of events needs further clarification; however, it is likely that AcrA and AcrB exist as a preformed complex and remain associated during the TolC docking (Zgurskaya and Nikaido, 2000). Upon opening of the periplasmic gates of TolC, as a result of the interaction between the crown of AcrB and the tip of the periplasmic end of TolC (Tamura et al., 2005), the H7/H8 helices of TolC partially relax and an intraprotomer groove is exposed in TolC, which forms part of the binding site for AcrA (Figure 3B). In the resting state, this groove is less pronounced, and the AcrA cannot be accommodated (Figures 2A and 2B), in accord with binding data (Touze et al., 2004). The engagement of AcrA into the intraprotomer groove in the partially opened state of TolC is likely to be required to enforce further opening of the channel (Figure 3C). Communication of the signal for opening of TolC likely arises from an AcrA/AcrB interaction. Thus, the activation of AcrB drives a conformational switch that affects both the destabilization of the TolC gates and the presentation of AcrA, and the latter becomes buried at the intraprotomer groove, transferring the energy provided by AcrB and driving the full opening of the TolC channel. Given the asymmetry of AcrB and, as indicated here, the TolC as well, it seems likely that the opening of the TolC channel in efflux pumps involves an energy-dependent, dynamic mechanism dependent on the sequential interplay of all three components.

## Experimental Procedures

### Antibiotic Sensitivity Studies of AcrA and TolC Point Mutants

For the antibiotic sensitivity studies of point mutant, TolC and AcrA were expressed from pTrc99A and pBAD33 plasmid backbones, respectively, in a background in which the chromosomal *tolC* and *acrA* genes have been deleted. Background levels of expression from pTrc99A were used, as it has been established that the pTrc promoter produces proteins at levels similar to that expressed from the chromosomal copy. AcrA expression from the pBAD33 promoter was induced by 0.2% arabinose w/v. Antibiotic sensitivities were determined by measuring inhibition zones around paper disks (6.5 mm diameter) soaked with either 30 μg novobiocin or 75 μg vancomycin. Plates were incubated for 8 hr at 37°C. Averages from two or more independent experiments were determined, with values varying no more than 10%.

### Cloning, Mutagenesis Expression, and Purification

For the TolC double mutant (Y362F,R367E) a fragment containing residues 1–452 of WT TolC (EMBL AE000385) was cloned into NdeI/XhoI sites of pET41b, so 8xHis tag from the vector is separated by L-E linker from the final residue of TolC (position 452). The C-terminal 43 residues are missing in this construct to favor growth of crystals. Deletion of the tail has no effect on in vivo activity (data not shown). Y362F and R367E mutants were introduced using the QuikChange Site-Directed Mutagenesis Kit (Stratagene). C41(DE3) and C43(DE3) (Lucigen Corp.) were expression hosts. Protein purification is described in detail in the Supplemental Data.

### Crystallization

Crystals were grown by vapor diffusion from hanging droplets. The orthorhombic (P2_1_2_1_2_1_) form was grown by mixing equal amounts of protein in storage buffer and reservoir solution containing 30% PEG 400 and 100 mM CAPS (pH 10.5) (10.5 mg/ml protein concentration). The monoclinic (C2) form was grown using a reservoir solution containing 0.1 M HEPES (pH 7.5), 5% isopropanol, 10% PEG 4000. Crystals appeared after 2–5 days (P2_1_2_1_2_1_) or 3–4 months (C2).

### Data Collection, Model Building, and Refinement

Crystals of the P2_1_2_1_2_1_ form were flash frozen into liquid nitrogen directly, while the C2 crystals were first cryoprotected using gradual transfer into 30% PEG 400. Data sets from C2 and P2_1_2_1_2_1_ crystals were collected at beamline ID23-2 of the European Synchrotron Radiation Facility (Grenoble, France) at λ = 0.8726 Å. Diffraction images were processed with DENZO and SCALEPACK (Otwinowski and Minor, 1997), and data were reduced using Truncate (CCP4, 1994). The orthorhombic form has pseudotetragonal symmetry, and a self-rotation function revealed the presence of a noncrystallographic trifold.

The merged, indexed, and scaled data from both sets of crystals were phased by molecular replacement using PHASER (Storoni et al., 2004) with a monomer of the wild-type TolC as the search model (1EK9). Table 2 summarizes the data, and details of the model building are described in the Supplemental Data.

### Rigid-Body Docking and Energy Calculations

Rigid-body docking and scoring were computed using pyDock (Man-Kuang Cheng et al., 2007). Details of the protocols are described in the Supplemental Data.

## Figures and Tables

**Table 1:** Antibiotic Sensitivities of the TolC Variants

	Evidence that the Mutant Makes the Outer Membrane Leaky without Affecting Efflux Pump Assembly: Novobiocin/Vancomycin Inhibition Zones with Complementation (in mm)		
Strain	None^a^	TolCΔ43a,b	TolCΔ43,a,b Y362F/R367E
Δ*tolC acrA*^+^*acrB*^+^	16.6/7.4	11.3/8.5	13.0/16.5
Δ*tolC* Δ*acrA acrB*^+^	17.8/7.5	16.5/8.5	17.8/16.3
Δ*tolC acrA*^+^ Δ*acrB*	15.8/7.4	17.0/8.8	18.8/17.0

	Evidence for Revertant Phenotype in Efflux Pump Activity: Novobiocin Inhibition Zones (in mm)		

Protein^c^			
TolC-WT, AcrA-WT	9.0		
TolC K383D, AcrA-WT	11.6		
TolC-WT, AcrA-D149K	11.2		
TolC-K383D, AcrA-D149K	7.8		

	Evaluation of TolC Leakiness: Vancomycin Inhibition Zones (in mm)		

TolC-WT, AcrA-WT	8.1		
ΔTolC, AcrA-WT	6.8		
TolC K383D, AcrA-WT	7.6		
TolC-R390E, AcrA-WT	16.0		

a Antibiotic sensitivities were determined by measuring inhibition zones around soaked paper disks (6.5 mm diameter). Values are in millimeters and are given for novobiocin on the left of the dash and vancomycin to the right. Averages from two independent experiments are shown, with values varying no more than 10%.

b A construct with the C-terminal 43 residues removed was used to favor growth of crystals (Koronakis et al., 2000); deletion of the tail has no effect on in vivo activity.

c Measurements were made at least in duplicate and vary by no more than 10%.

**Table 2:** Crystallographic Data and Refinement of the Y362F, R367E Mutant TolCΔ43 Structures

	TolC Y362F,R367E—C2	TolC Y362F,R367E—P2_1_2_1_2_1_
Data Collection		

Space group	C2	P2_1_2_1_2_1_
Cell dimensions		
a (Å)	122.49	128.28
b (Å)	70.97	136.18
c (Å)	219.95	136.13
β (°)	100.61	
Resolution (Å)^a^	3.30 (3.35−3.30)	3.20 (3.30−3.20)
Crystals	6	6
R_merge_ (%)	14.7 (39.8)	15.2(42.1)
I/σ_I_	7.46 (2.45)	9.53 (2.45)
Completeness (%)	98.00 (99.1)	97.7 (98.3)
Redundancy	3.3 (3.3)	5.0 (5.0)

Refinement		

Resolution (Å)	29.80−3.30	22.1−3.20
	(3.36−3.30)	(3.30−3.20)
Number of reflections	26,721 (1773)	36,034 (2216)
R_work_	23.6 (29.1)	26.6 (36.7)
R_free_^b^	29.5 (42.4)	31.5 (41.6)
Number of atoms used in refinement	9869	9734
B factors		
Protein (Å^2^)	48.2	67.8
Rmsd		
Bond lengths (Å)	0.007	0.007
Bond angles (°)	0.979	1.033

All data were collected at wavelength 0.8726 Å. Because of the low resolution (3.2 and 3.3 Å for the orthorhombic and monoclinic forms, respectively), no water molecules were placed in the models apart from the ones in the putative chloride-binding pockets, where it was possible to locate them due to their precise coordination (Figure S1).

a Highest resolution shell is shown in parentheses.

b Five percent of the reflections were set aside for calculation of the R_free_ value.

## Accession Numbers

Coordinates have been deposited with the Protein Data Bank with accession codes 2VDD and 2VDE.

## Supplemental Data

Document S1. Three Figures, One Table, Supplemental Text, and Supplemental References
