# Supplementary material for: Assembly and Channel Opening in a Bacterial Drug Efflux Machine
Source: Mol Cell. 2008 Apr 11;30(1):114–21. doi: 10.1016/j.molcel.2008.02.015 (PMC2292822; doi:10.1016/j.molcel.2008.02.015)
Supplement: Document S1. Three Figures, One Table, Supplemental Text, and Supplemental References [file mmc1.pdf]

Molecular Cell, Volume *30*

Supplemental Data

Assembly and Channel Opening in a Bacterial Drug Efflux Machine

Vassiliy N. Bavro, Zbigniew Pietras, Nicholas Furnham, Laura Pérez-Cano, Juan Fernández-Recio, Xue Yuan Pei, Rajeev Misra, and Ben Luisi

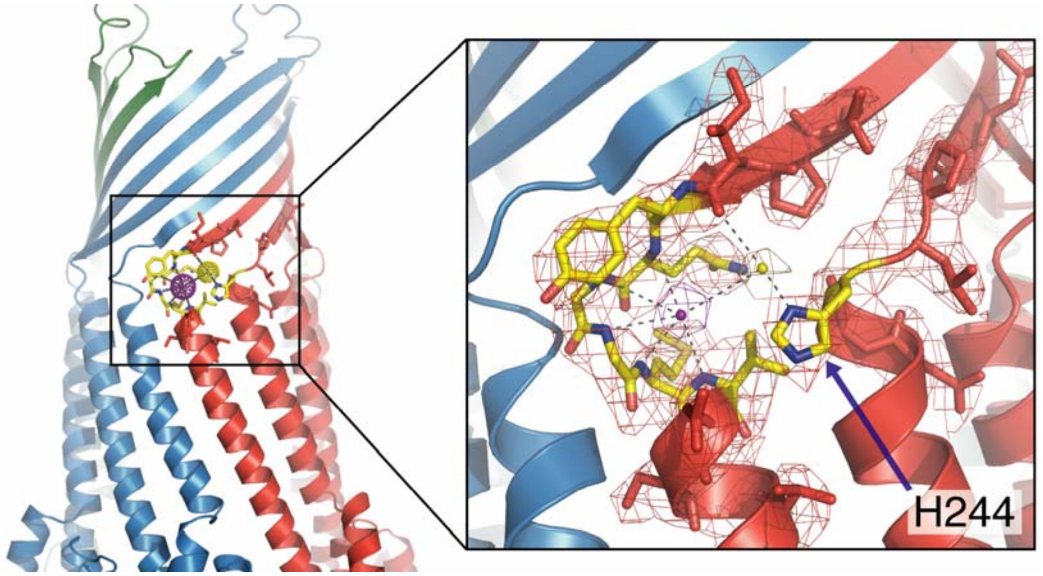

Figure S1

A putative chloride binding site in TolC.

The C2 structure, viewed at the junction of the transmembrane and helical subdomains. The purple dotted sphere indicates a putative chloride that interacts with the exposed amide groups at the end of the α-helix. A water molecule is also found in the pocket (yellow dotted sphere) supported by interaction with a histidine imidazole. The mesh represents the 2Fo-Fc electron density map contoured at 1σ. Similar density in the corresponding position is observed in the $P2_12_12_1$ form. Anion binding is likely to serve purely a structural role and appears to be a unique feature of the TolC channel, not present in the crystal structure of the homologous VceC and OprM outer membrane proteins.

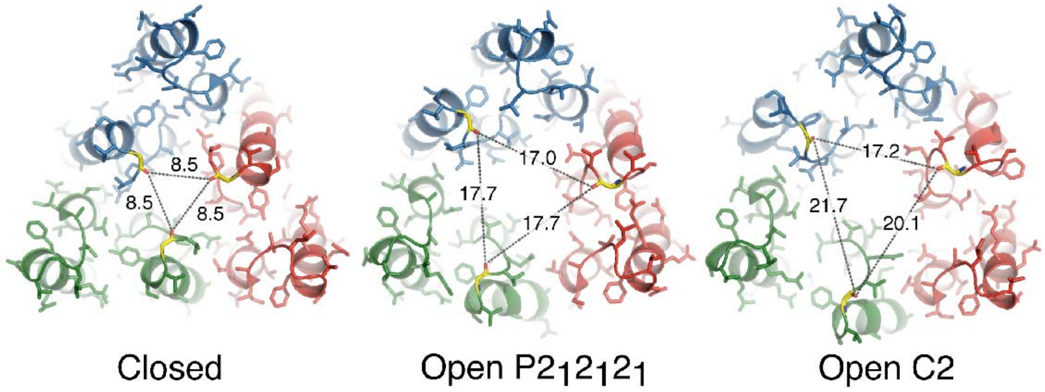

Figure S2

Asymmetry of the TolC open state.

Opening and asymmetry of the periplasmic end of the TolC channel as observed in the available crystal structures. The subunit non-equivalence in the crystal structure is most clearly seen in the inter-protomer distances. The outer rim of the TolC channel as defined by G365 is opening up in both novel crystal forms as compared with the closed state. The displacement of this residue also indicates clearly the asymmetric opening of the channel.

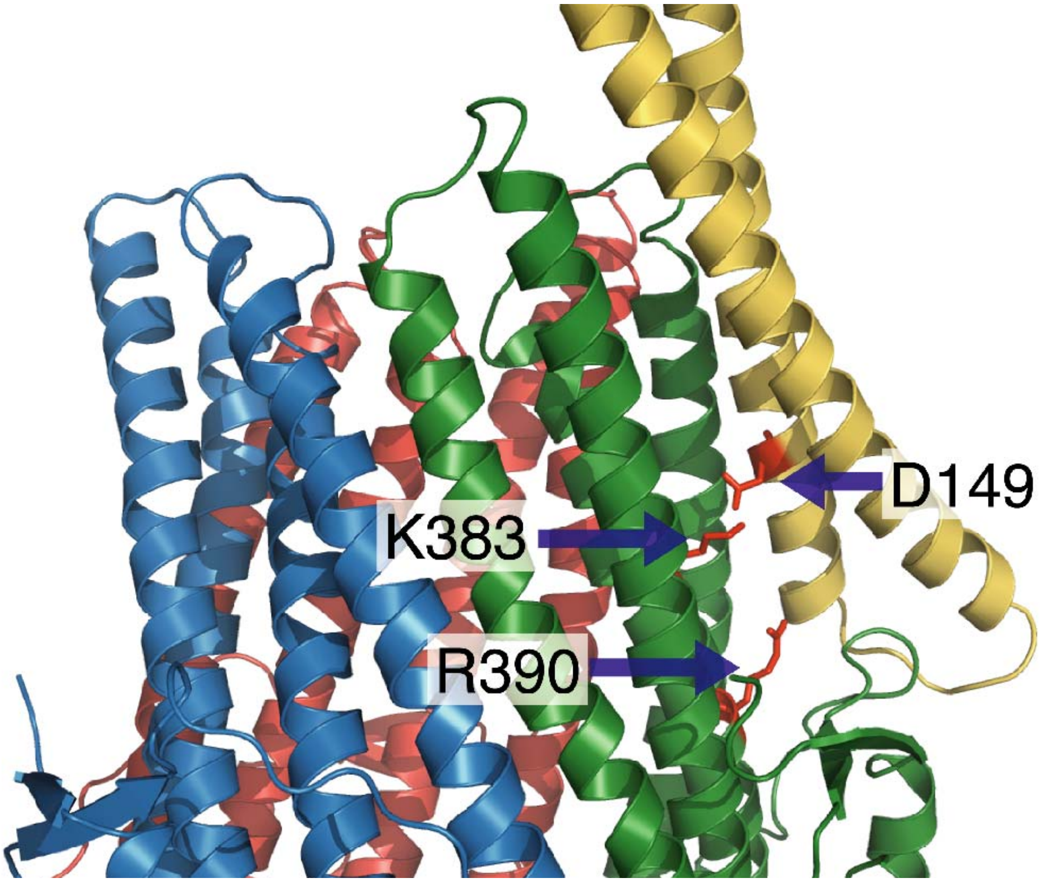

Figure S3

A docking model of the interaction of the AcrA hairpn (yellow) with TolC C2 structure.

The residues highlighted in red include K383 and R390 from TolC and D149 from AcrA. The numbering of AcrA is according to the full-length sequence of the protein as represented in the 2F1M PDB entry.

Protein purification

C41(DE3) and C43(DE3) cells were grown in 2xYT media at 37°C, induced at OD_{600} of roughly 0.6, and then were grown for 12 hours at 24°C. Cells were harvested in lysis buffer (20 mM Tris-Cl pH 8.0, 150 mM NaCl, 20 mM $MgCl_2$, complete EDTA free proteinase inhibitor cocktail (Roche), supplemented with DNase I and lysozyme), lysed using an Emulsiflex C5 cell homogenizer (Avestin, Canada) and cell pellet removed by centrifugation at 10,000 x g. The membrane fraction from the supernatant was pelleted by centrifugation at 100,000 x g for 3 hours. The membranes were solubilized in 20 mM Tris-Cl pH 8.0, 150 mM NaCl, 20 mM $MgCl_2$, 2.5 mM $CaCl_2$, 2.5 mM KCl, 2% v/v Triton X100 on a rotary shaker at 4°C overnight, centrifuged at 100,000 x g for 2 h and supernatant diluted 4 fold in column equilibration buffer (20 mM Tris-Cl pH 8.0, 150 mM NaCl, 0.15% v/v Triton X100 and 5 mM imidazole) and applied to a Ni-Chelating column (HiTrap, Amersham Biosciences). Protein was eluted with a linear gradient to 400 mM imidazole. TolC enriched fractions were applied on to anion exchange column (HiTrapQ, Amersham Biosciences), over which the detergent was exchanged to 0.05% (w/v) βDDM. Protein was eluted with a salt gradient and applied to an S200 size-exclusion column (Pharmacia) to transfer to storage buffer (20 mM Tris-Cl pH 8.0, 100 mM NaCl, 0.05% w/v βDDM).

Crystallographic model building

In the regions of structural movement, sections of the model were manually fitted into the electron density as rigid bodies. The poorly fitting sections as identified by real-space scoring function as implemented in RAPPERtk (Gore et al., 2007) and regions of poor geometry as identified by MolProbity (Davis et al., 2007), were rebuilt using RAPPERtk following the protocol used by RAPPER for low resolution model building (Furnham et al., 2006). The resulting models were refined using CNS (Brunger et al., 1998) and manually built in COOT (Emsley and Cowtan, 2004). In the final stages, REFMAC 5.3 was used (Murshudov et al., 1997). Residues after 428 are disordered in the structure. Model and refinement statistics are presented in Table 2. Figures were made using PyMol (http://pymol.sourceforge.net/). Secondary structures were assigned using DSSP (Kabsch and Sander, 1983). Ramachandran statistics for the refined structures were determined using PROCHECK (Laskowski et al., 1993) and RAMPAGE (Lovell et al., 2003)

Rigid-body docking

We applied a recently described protocol for rigid-body docking and scoring: pyDock (Man-Kuang Cheng et al., 2007). A total of 10,000 rigid-body docking poses were generated by FTDOCK 2.0 with no electrostatics (thickness 1.3 Å, grid size 1.2 Å)(Gabb et al., 1997). These docking poses were evaluated by a scoring function that included: *i*) Coulombic electrostatics with distance-dependent dielectric constant ($\varepsilon=4d$), atomic charges from AMBER 94 force field (Cornell et al., 1995) and pairwise interaction energy values truncated to a maximum and minimum of +1.0 and -1.0 kcal/mol, respectively; and *ii*) effective water-to-interface desolvation energy based on atomic accessible surface area (*ASA*), with atomic solvation parameters (*ASP*s) optimized for rigid-body docking (Fernandez-Recio et al., 2004; Fernandez-Recio et al., 2005). For the docking of TolC with AcrA, we defined distance restraints from cross-linking data (Lobedanz et al., 2007) on residues in TolC (S124, Q139, Q142, S363) and AcrA (A99, A103, D111, L124, R128, Q136, I138, E142), which were added to the final scoring function with pyDockRST module (Chelliah et al., 2006).

Docking models for TolC/AcrB interaction were refined using *ad-hoc* protocols. For the two possible orientations we obtained, different conformations were generated by randomly sampling (within a maximum of 5 degrees) the mutual rotation of both molecules TolC and AcrB, together with random sampling (within 5 Å) of the intermolecular distance, while keeping the trimeric symmetry axis. These docking orientations were later refined by interface side-chain sampling with SCWRL, (Canutescu et al., 2003) and the one with the best pyDock (Man-Kuang Cheng et al., 2007) energy was selected.

Accessible surface calculations were performed using AREAIMOL v6.0 (CCP4). Molecular volume calculations performed using CASTp server and evaluated using CASTp plug-in for PyMol (Binkowski et al., 2003).

Optimal Docking Area calculations

Optimal Docking Areas (ODA) of unbound TolC, AcrA, and AcrB, were calculated following a variation of a previously described method (Fernandez-Recio et al., 2005). For each surface residue, we computed the desolvation energy based on accessible solvent area for a surface patch formed by itself and the surrounding residues. Several distance values *d* (*d* = 1, 2, …, 20 Å) were used to find the surface patches with the best desolvation energy. Residues with patch desolvation energy < -10.0 kcal/mol define regions on the protein surface that are likely to be involved in protein-protein interactions.

Table S1 Docking energies for TolC/AcrB

Docking and refinement of asymmetric AcrB structure (2GIF.pdb) to a model of WT open state based on the C2 crystal form of TolC.

Model [a]	Binding energy (kcal/mol) [b]	Satisfied restraints [c]	
		TolC	AcrB
Orientation 1	-51.4	G147	Q255,D256,G257
		A150	Q255
		R143	Q255
		G365,T366	D795,G796
		R367	D795
Orientation 2	-18.7	G147	Q255,G257
		Q142,R143	Q255,D256
		G365,T366	D795,G796
		R367	D795

[a] Refined docking models for TolC:AcrB interaction. Orientation 1 is the same as in Figure 3B. Orientation 2 is an alternative low-energy rotation of AcrB and TolC along the three-fold symmetry axis.

[b] Binding energy of the best refined model for each orientation is evaluated with pyDock (van der Waals + electrostatics + desolvation)

[c] Satisfied restraints in the model are defined by those residue pairs whose Cα atoms would be at $<$ 10Å, as expected from cross-linking experiments (Tamura et al., 2005).

SUPPLEMENTAL REFERENCES

Binkowski, T. A., Naghibzadeh, S., and Liang, J. (2003). CASTp: Computed Atlas of Surface Topography of proteins. Nucleic Acids Res *31*, 3352-3355.

Brunger, A.T., Adams, P.D., Clore, G.M., DeLano, W.L., Gros, P., Grosse-Kunstleve, R.W., Jiang, J.S., Kuszewski, J., Nilges, M., Pannu, N.S., Read, R.J., Rice, L.M., Simonson, T. and Warren, G.L. (1998) Crystallography & NMR system: A new software suite for macromolecular structure determination. Acta Crystallographica Section D-Biological Crystallography, 54, 905-921.

Canutescu, A.A., Shelenkov, A.A. and Dunbrack, R.L., Jr. (2003) A graph-theory algorithm for rapid protein side-chain prediction. Protein Sci, 12, 2001-2014.

Chelliah, V., Blundell, T.L. and Fernandez-Recio, J. (2006) Efficient restraints for protein-protein docking by comparison of observed amino acid substitution patterns with those predicted from local environment. J Mol Biol, 357, 1669-1682.

Cornell, W.D., Cieplak, P., Bayly, C.I., Gould, I.R., Merz, K.M., Ferguson, D.M., Spellmeyer, D.C., Fox, T., Caldwell, J.W. and Kollman, P.A. (1995) A 2nd Generation Force-Field for the Simulation of Proteins, Nucleic-Acids, and Organic-Molecules. Journal of the American Chemical Society, 117, 5179-5197.

Davis, I.W., Leaver-Fay, A., Chen, V.B., Block, J.N., Kapral, G.J., Wang, X., Murray, L.W., Bryan Arendall, W., 3rd, Snoeyink, J., Richardson, J.S. and Richardson, D.C. (2007) MolProbity: all-atom contacts and structure validation for proteins and nucleic acids. Nucleic Acids Res.

Emsley, P. and Cowtan, K. (2004) Coot: model-building tools for molecular graphics. Acta Crystallographica Section D-Biological Crystallography, 60, 2126-2132.

Fernandez-Recio, J., Totrov, M. and Abagyan, R. (2004) Identification of protein-protein interaction sites from docking energy landscapes. J Mol Biol, 335, 843-865.

Fernandez-Recio, J., Totrov, M., Skorodumov, C. and Abagyan, R. (2005) Optimal docking area: a new method for predicting protein-protein interaction sites. Proteins, 58, 134-143.

Furnham, N., Dore, A.S., Chirgadze, D.Y., de Bakker, P.I., Depristo, M.A. and Blundell, T.L. (2006) Knowledge-based real-space explorations for low-resolution structure determination. Structure, 14, 1313-1320.

Gabb, H.A., Jackson, R.M. and Sternberg, M.J. (1997) Modelling protein docking using shape complementarity, electrostatics and biochemical information. Journal of Molecular Biology, 272, 106-120.

Gore, S.P., Karmali, A.M. and Blundell, T.L. (2007) Rappertk: a versatile engine for discrete restraint-based conformational sampling of macromolecules. BMC Struct Biol, 7, 13.

Kabsch, W. and Sander, C. (1983) Dictionary of protein secondary structure: pattern recognition of hydrogen-bonded and geometrical features. Biopolymers, 22, 2577-2637.

Laskowski, R.A., MacArthur M.W., Moss D.S. & Thornton J.M. (1993) PROCHECK: a program to check the stereochemical quality of protein structures. J. Appl. Cryst., 26, 283-291.

Lobedanz, S., Bokma, E., Symmons, M.F., Koronakis, E., Hughes, C. and Koronakis, V. (2007) A periplasmic coiled-coil interface underlying TolC recruitment and the assembly of bacterial drug efflux pumps. Proc Natl Acad Sci U S A, 104, 4612-4617.

Lovell, S. C., Davis, I. W., Arendall, W. B., 3rd, de Bakker, P. I., Word, J. M., Prisant, M. G., Richardson, J. S., and Richardson, D. C. (2003). Structure validation by Calpha geometry: phi,psi and Cbeta deviation. Proteins *50*, 437-450.

Man-Kuang Cheng, T., Blundell, T.L. and Fernandez-Recio, J. (2007) pyDock: electrostatics and desolvation for effective scoring of rigid-body protein-protein docking. Proteins, 68, 503-515.

Murshudov, G.N., Vagin, A.A. and Dodson, E.J. (1997) Refinement of macromolecular structures by the maximum-likelihood method. Acta Crystallogr D Biol Crystallogr, 53, 240-255.

Tamura, N., Murakami, S., Oyama, Y., Ishiguro, M. and Yamaguchi, A. (2005) Direct interaction of multidrug efflux transporter AcrB and outer membrane channel TolC detected via site-directed disulfide cross-linking. Biochemistry, 44, 11115-11121.
